# Hospital Meetings, &c.

**Published:** 1900-06-02

**Authors:** 


### HOSPITAL MEETINGS, &C.

### HOMES FOR WORKING BOYS IN LONDON.

The triennial festival of the Homes for Working Boys in London was held on Monday evening, the 21st ult., at the Queen's Hall, Langham Place. The proceedings opened with a dinner to the 340 boy residents of the seven homes occupied and carried on by the committee, of whom the Earl of Aberdeen is the president. Unfortunately his lordship was unable to be present at what proved to be a singularly joyous function, for much *éclat* was added to the festival by the stimulating presence of Sir George White, V.C., who was received on his appearance with deafening cheers and the waving of flags and handkerchiefs. During the progress of the dinner to the boys the large hall rapidly filled with subscribers and friends of the institution, and, the meal being over, Lord Kinnaird presided over the after-meeting. Among those on the platform in addition to Sir George White were: Lord Chelmsford, Sir Douglas Fox, Mr. Quintin Hogg, Mr. Samuel Hope Morley, Mr. T. A. Denny and Lady Hope, the treasurer and honorary secretary (Mr. T. H. W. Pelham), and the Dean of Norwich. The object of the meeting, it was explained, was to raise funds for the acquisition and fitting up of the new home in Queen Square, W.C., the amount necessary for these purposes being put at £1,500, and in addition the committee desired another £1,000 for the general maintenance of the work. Mr. Pelham very humorously pleaded for aid, and trusted to receive the contributions of friends, who had merely to address them to "Pelham, Putney." Lord Kinnaird having delivered a short speech, Dean Lefroy addressed the gathering in rousing terms and advised the boys to always bear in mind and strictly practise the "three P.'s"—Pluck, Perseverance, and Prayer.

The rising of Sir George White was the signal for another outburst of vigorous cheering, and he was unable to speak for some minutes. In a few happily chosen phrases Sir George said he thought that if anyone had a right to describe himself as a "skeleton at the feast" it was surely the latest arrival from Ladysmith. Referring to the work of the homes, he said he looked upon such training of boys as an immense advantage to the Service, to which he felt the greatest pride in belonging. He hoped that many of the lads would do what was the first duty, to serve their country in her hour of need. When he (the speaker) first joined the army there was an idea that a reckless, lawless man was the material for the best soldier. It was said that such a man would "come out" when there was a real fight. It was true there was a certain recklessness, combined with a certain breaking of laws, which carried such men a certain distance; but when men were half fed and the nights were cold and wet, and men's nerves were broken down by shot and shell, the lawless fellow disappeared. It was when he was called upon to replace some poor comrade shot on picket that the man who turned out and took his place proved to be the man who had disciplined himself and did what his Queen and his country expected him to do from a sense of duty.

### PADDINGTON GREEN CHILDREN'S HOSPITAL.

Mr. George Hanbury, the treasurer, presided at the twentieth annual meeting of the Paddington Green Children's Hospital on the 24th ult. The report was read by the hon. secretary, Mr. C. W. Empson. The committee stated that they had continued through the year the policy pursued in 1898 of strengthening their financial position and improving the general work of the hospital. The debt to their bankers had been reduced from £1,300 to £1,000; annual subscriptions had somewhat increased; and the sums received from the Hospital Saturday and Sunday Funds were increased by 18 guineas and £110 respectively, the amounts being £101 and £260. The Prince of Wales's Fund had again sent £150. The ordinary income was £3,274 and the ordinary expenditure £3,651. The extraordinary income was £2,671, and the extraordinary expenditure £1,228. The passenger lift, for which special subscriptions were invited, had been completed and was now in full use, to the great advantage both of patients and of staff. The cost was £396, and only a small balance—some £14—remained to be collected. Acting on the recommendation of the medical staff, a Röntgen Ray apparatus had been purchased at a cost of just over £80. The supervision of this had been undertaken by Dr. Alexander Low, and it had already been of much use. Another improvement had been the connection of the hospital with the National Telephone system, thus ensuring speedier communication with the staff as well as with the Metropolitan Asylums Board in dealing with infectious cases. £277 had been contributed towards the liquidation of the building fund debt as the proceeds of a Café Chantant at the Paddington Baths; and thanks were tendered to Miss Scott and other ladies who had organised and carried out the arrangements. Others to whom gratitude was expressed were the Lancaster Gate Club, the pupils of Miss Manville, and the Southall Cycle

Clubs, who contributed £18, £40, and £10 respectively. The committee noted with gratification that in spite of the many demands on the public, the congregational collections showed scarcely any falling off. Among these Christ Church, Lancaster Gate, was as usual foremost, contributing £218. The convalescent home continued to be of the utmost use to the hospital; 84 children had passed through it during the past year, but the committee expressed their regret that so little support was given to it. Including £50 from the Prince's Fund, the receipts only amounted to £211, while the expenditure was £483. The number of in-patients admitted during 1899 was 595; the new out-patient cases numbered 12,873, with total attendances of 43,453; the casualties and accidents being 1,155.

In moving the adoption of the report and accounts, the Chairman reminded the meeting that the hospital was no longer a new one. It was five years since they had been in the present building, and twenty years ago since their first opening. Their finances were not in a very bad way, but they still owed £1,000 to their bankers. He did not wish any of their friends to die, but he did wish some good legacies would fall in. As the report stated, their lift was very nearly paid for, and now they badly wanted a new refrigerator. He hoped a special effort would be made to supply this. He paid a warm tribute to Miss Anderson, the matron, whose services were invaluable.

Mr. F. D. Mocatta seconded the motion, which was carried *nem. con.*

General R. W. Lowry moved the re-election of the retiring members of the committee; and votes of thanks brought the proceedings to a close.

### EAST LONDON HOSPITAL FOR CHILDREN.

### Festival Dinner.

The festival dinner in connection with the East London Hospital for Children, Shadwell, was held at the Hotel Cecil on Friday evening, May 25th. Mr. Hugh Colin Smith presided over a large company of ladies and gentlemen, including Lord and Lady Falkland, the Hon. Catherine Cary, Lady Maud Wilbraham, Lady Louisa Magenis, the Hon. Mrs. Bevan, Alderman Sir J. Whittaker Ellis and Lady Ellis, Mr. W. H. Dickinson, L.C.C., Mrs. and Miss Dickinson, Mr. H. W. Trinder, Mr. and Mrs. Charles Cheston, Mr. Vivian H. Smith, Miss Crawfurd, Major Roper Parkington, Mr. C. F. Bellamy (master of the Poulters' Company), and Dr. J. A. Coats.

After the customary expressions of loyalty,

The Chairman submitted "Prosperity to the East London Hospital for Children." A hospital for children, he remarked, was an institution which required little to recommend it from anyone in his position, for the mere mention of children at once appealed to all, while the mention of children whose homes were so poor that they were deprived of medical comforts therein, and of the good food which their circumstances required, should appeal to all beyond anything that words could convey. (Hear, hear.) He had on the previous Wednesday had the pleasure of visiting the hospital, and could only say that if his hearers would follow his example in that matter they would be, as he had been, thoroughly satisfied with all they saw. It was in every sense of the word a hospital up to date, and the fact that ladies were present at this festival dinner was some evidence, at any rate, that that was so. Its squalid surroundings notwithstanding, the hospital was light and cheerful and the children were happy. The little ones were well supplied with toys, and everything that could be done to alleviate their sufferings was done. A visitor to the hospital could not but feel that a great work had been undertaken there, in a district where there was sore need for it. There was every reason why it should be well supported, and the support that was particularly required was the replacing of subscribers as they died or otherwise ceased their financial help. There were, he was informed, several matters that required attention just now. Of course, to a hospital in a district like Shadwell, where the air was none of the freshest, a convalescent home was a great boon, and the home at Bognor, which was part and parcel of the work of this institution, was therefore deserving of cordial help. It had, as a matter of fact, drained the funds of the hospital to the extent of £300 a year, and it would be a great relief to the authorities if that could cease. Such an adjunct, the importance of which it was impossible to exaggerate or over-estimate, should not be allowed to be a burden upon the parent institution. He sincerely trusted, too, that the £8,000 which was required for the purpose of carrying out absolutely necessary improvements at Shadwell would be soon forthcoming. It was a large sum, but his experience of Englishmen was that if they had confidence in an institution they did not keep it waiting long for the financial support of which it stood in need. He understood that a ladies' association had been recently formed in connection with the hospital—(applause)—and he was certainly glad to see ladies taking part in a work for which they were so eminently fitted. In appealing for funds, the Chairman mentioned the rise in prices which has taken place throughout the country generally, and he concluded by saying that he looked with the greatest satisfaction upon the way in which the institution had been started and managed. (Applause.)

Mr. H. W. Trinder, in acknowledging this toast, as he said, for the ninth time, bore grateful testimony to the fact that, although the hospital is situated in a place where hardly anyone saw it unless they purposely visited it, it is well supported by friends. He alluded to the valuable assistance given to the hospital by the City, and particularly by the livery companies. So far as the annual income and expenditure was concerned, they usually went on very well, and were able to avoid running into debt. It was when they came to any special expenditure that they experienced some temporary difficulty in meeting it. As an instance, he mentioned the £8,000 which is now required. When, however, they made their wants known they were able in the course of a short time to get over their difficulties. In the past 30 years they had spent about £50,000 in the purchase of land at Shadwell and Bognor and in the erection of buildings. It was no exaggeration to say that many of the children passed their happiest days when they were in the hospital, for the bulk of them came from families who lived in one room. During the past year their out-patients increased in numbers, but he was glad to say the in-patients showed a slight diminution. This latter fact was due to there being fewer children coming to the hospital suffering more or less from starvation, the comparative prosperity of the working men of the country doubtless, and the consequent better feeding of the children, being the explanation of this. In the course of the year they had various calls upon their income for capital purposes, but they managed to end the period with only £142 more expended than received. This deficit arose in consequence of their spending something like £1,000 in building a new operating theatre and a small additional ward for surgical cases—which would be open about a month or six weeks hence—and also in meeting certain requirements in the home at Bognor. There was still something further required in the convalescent home—a laundry and an isolation ward—but their friend, Mr. Prescott, had kindly given them the £550 which was required for these purposes. Then for the out-patients—who had increased in number from 3,000 in 1875 to 36,000 in 1899—they required further accommodation; the same might be said of the resident staff and nurses; and they also wanted an enlarged kitchen. Then it had become necessary to have a permanent casualty department near the dispensary, and there were many uses to which they could put their temporary casualty department. For these improvements some £6,000 was required, and rebuilding the laundry and erecting a new mortuary and examination room would bring the sum needed up to £8,000. If a good start were made towards this that night he thought they might be successful in raising the rest of the money in the course of twelve months. The hospital would thus be enabled to cope with the increasing work which was demanded of it. Concluding, Mr. Trinder urged that, while the charitable were giving in new channels, old claims should not escape their attention.

In giving "The Board of Management and the Medical Staff," Sir Whittaker Ellis eulogised the services of those comprised in the toast, and proceeding to refer to the claims of the hospital he said he looked with disapproval upon the provision of war funds as a matter of charity. Such funds should be provided by the country. Our soldiers fought for their country, and the country should pay what was necessary. The country ought to be taxed, and if it were it would pay cheerfully to provide for the families of the men who were killed in the war. Our soldiers were entitled, not to charity but to their just rights, and although he had supported many of the war funds by both labour and purse, he was of opinion that the time had come when he and those who thought as he did ought to speak out and tell the Government that it was their duty to provide for the families of those soldiers whose lives were given in the service of their country.

Mr. Charles Cheston, the treasurer, whose name was associated with the toast, briefly replied, claiming that this institution was undoubtedly the best of the children's hospitals in London. While it was as efficient as any, it was carried on at much less cost per cot than any similar hospital in the metropolis.

"The Ladies and Visitors," given by Mr. W. A. W. Scott and replied to by Mr. W. H. Dickinson, and "The Chairman," proposed by Mr. Trinder, were the other toasts.

### NORTH-EASTERN HOSPITAL FOR CHILDREN.

Mr. Herbert Robertson, M.P., presided at the thirty-second annual meeting of governors of the North-Eastern Hospital for Children, Shoreditch, held on Thursday, May 24th.

The Secretary (Mr. T. Glenton-Kerr) read the report of the general committee, which stated that during the past year the number of children who received the benefits of the hospital were: In-patients, 748; out-patients, 16,665; and the number of attendances of the latter, 59,203. The accounts showed that the ordinary income of the hospital for 1899 amounted to £6,110, and the extraordinary to £100, a total of £6,210. The ordinary expenditure was £5,743, and the extraordinary (building improvements, fittings, electric light installation, and surgical instruments) £86, in all, £5,829. There is consequently a balance of £381 on the working of the hospital for the year, from which falls to be deducted a debit balance of £24 on the convalescent fund, the income of which was £70, and the expenditure £94. The remaining £357 is applied in reduction of the deficit of £385 brought forward, and the adverse balance now stands therefore at only £28. The total receipts (£6,280) were £143 more, and the total expenditure (£5,923) £168 less, than in 1898. The committee point with particular satisfaction to the receipt of £500 from the Prince of Wales's Hospital Fund as an annual subscription, the contributions of the two previous years having taken the form of donations. This fund also made a special grant of £250 to the building fund. In view of the large increase of work contemplated in the immediate future, the committee invite the co-operation of all those interested in the hospital in an endeavour to increase the annual subscriptions by at least £1,000 per annum. The legacies received during the year, amounting to £850, have been placed to the building fund account. The total receipts on this account, including £3,152 realised on sale of North-Eastern Railway stock, were £5,243, and the expenditure £7,560, leaving an adverse balance of £2,317. Deducting this last item from the amount of £2,912 brought forward, there remains in hand £595. With an investment in North-Eastern Railway consols valued at, say, £2,500, and an amount of £2,061 promised, there is therefore available for building purposes £5,156. After careful investigation it was decided to purchase three houses in Hackney Road, adjoining the hospital, at a cost of £2,500. In order to facilitate the carrying out of the building scheme it has been decided to build the wards and other much-needed accommodation on the enlarged site facing Hackney Road thus obtained as soon as funds will allow, postponing for the present the erection of the new out-patients' department and nurses' home, to be placed on the Goldsmith Row site. The total cost of the entire scheme of building is estimated at £40,000, £25,000 of which, according to the architect's calculation, will complete the building to be first proceeded with on the Hackney Road site, while the remaining £15,000, it is roughly estimated, will cover the cost of the second contemplated premises already referred to. The expense of the scheme thus altered being much greater than anticipated at first, the committee decided to defer building operations until they had received promises of a larger sum than that thought necessary a year ago. The disturbed state of the national affairs prevented any attempt to raise the money for some months, but the committee now feel that further delay would not be justifiable, and they propose shortly to make an appeal which they hope will meet with the liberal response they think the object in view deserves. The report referred with special satisfaction to the successful working during the year of the system of inquiry into out-patient cases, commenced on June 1st, 1898; the absence of any friction between the inquiry officer and the parents, notwithstanding the searching nature of the investigation made in many cases, being particularly pleasing to the committee. Extended inquiries were made in 178 cases, the result of which was the classification of 89 cases as unsatisfactory, 12 as untraceable and only attended once, and the remaining 77 (including six first referred to a local practitioner for certificates of suitability) as satisfactory. The only change in the staff mentioned in the report was the resignation, owing to pressure of other work, of Mr. H. P. Dean, who had been one of the surgeons to the hospital since 1889, the vacancy being filled by Mr. Douglas Drew, F.R.C.S. Mr. Dean was subsequently elected a consulting surgeon. Mr. C. V. Burton was re-appointed resident medical officer for a further twelve months. The office of president remains open, and the matter is receiving the careful attention of the committee.

The Chairman, in moving the adoption of the report, said that it must be considered satisfactory. What he wished most to say was with regard to the building scheme, which the meeting would see necessitated a much larger amount than was previously anticipated. When the demands on every other hospital in London were getting greater and greater, it was not a matter for surprise that they also were constantly experiencing great difficulty through want of room. He did not propose appealing for the whole £40,000 at first, though, of course, if it could be obtained he should be very glad. What the committee hoped to get now was the first £25,000 with which the building in the Hackney Road, giving the accommodation most urgently required, could be separately proceeded with and completed. The carrying out of the second part of the scheme could be deferred for the present, although the accommodation which it would provide was also needed more and more. Efforts had been made during the year to obtain a member of the Royal family as president of the hospital, but it was a matter of great difficulty, and they had so far been unsuccessful, things remaining in the same position as last year. He was glad to draw attention to the statement in the report as to the successful working of the inquiry system. When it was remembered what opposition it had met with at its inception in London a few years ago, and the difficulties other hospitals had had in introducing the system, it was satisfactory to note that already it was working well at this hospital.

Mr. C. G. Port, the chairman of the Building Committee, seconded the motion, and after summarising the state of the building fund, which is referred to in detail above, said that the question of the best way to raise the funds required had had the earnest attention of the committee. They had considered the advisability of holding a festival or bazaar, or anything in that way, but thought it better in the present state of the country not to do so. The war had thrown them back certainly twelve months; so many demands upon people's pockets had arisen from it that they seemed to have little or nothing left for an appeal of this kind. If they appealed within the next month this condition would still apply, while if they left it over for three months, when in all probability the war would be over, they would find themselves in the holiday season, and after that every other hospital in London would be making a similar appeal. A full committee meeting would be held in a week or so, when the matter would be finally decided, and in all probability an appeal would be sent out in the next few weeks, which he could only hope would meet with every success.

Mr. Ames also supported the motion for the adoption of the report, which was carried unanimously.

In moving the re-election of the officers and committee, Mr. G. B. Barnard called attention to the small number of that body present, only three out of 30 or 40, including the *ex-officio* members. He thought that at an annual meeting of this kind, and especially at a time when they were making large appeals, more of the members of the committee should at least make an effort to put in an appearance.

Mr. Freeman and Mr. Hurlin followed on somewhat similar lines.

The Secretary and Mr. Port mentioned the many claims upon their colleagues' time, both in the way of business and for the frequent meetings of the committee and of the various sub-committees of the hospital, which, they could assure them, were always very fully attended indeed. In addition, three members were detained by illness.

The motion was adopted.

The usual votes of thanks to the staff and to the chairman for his services concluded the proceedings.

